# Observations on the Phenomena of the Circulation

**Published:** 1828-01

**Authors:** J. H. James


THE LONDON
### Medical and Physical Journal.

No. 347, VOL. LIX.] JANUARY, 1828. [No. 19, *New Series*

For many fortunate discoveries in medicine, and for the detection of numerous errors, the world is indebted to the rapid circulation of Monthly Journals; and there never existed any work, to which the Faculty, in Europe and America, were under deeper obligations, than to the Medical and Physical Journal of London, now forming a long, but an invaluable, series.—RUSH.

### ORIGINAL PAPERS,

AND

### CASES OBTAINED FROM PUBLIC INSTITUTIONS AND OTHER AUTHENTIC SOURCES.

### THE CIRCULATION.

*Observations on the Phenomena of the Circulation.*

By J. H. James, [redacted].

There is no one subject in physiology which has at all times attracted so much attention as this; and convinced as I am that it has almost been pursued to satiety, I would not now offer any additional remarks, if I were not persuaded that the explanations given are still very unsatisfactory, and that circumstances of important influence in the consideration of the question have been overlooked.

The limits of this paper will necessarily be brief: nevertheless, it must be my object to review, in as concise a manner as I can, the subject generally; for any partial consideration of detached parts or circumstances can only be productive of error.

The flow of blood through the arteries is first to be considered. These vessels constitute a series of pipes, always full, but only occasionally distended. At each impulse of the heart, they receive an additional quantity, which at the moment urges a portion of that contained in them into the minute vessels, and also causes them to dilate, which their elasticity allows; the impulse having ceased, then elasticity reacts on the contained blood, and expels the remainder of the surplus portion which has been received, so that directly at the systole, and indirectly through the agency of this elasticity, the heart is, in point of fact, every moment of time employed in propelling the blood. A jet is perceived in the large branches at the systole, but in arteries of less size than one-sixth of a line in diameter the stream appears uniform, which may be explained by the velocity of the blood sent by the direct impulse being so much retarded in its progress, that the velocity of the steam, as maintained by the elastic power, is hardly exceeded by it, and scarcely any difference can be perceived in the uniformity of the current: at least, such appears to have been the opinion of Mr. Hunter.

This doctrine has been assailed by many able physiologists, but more particularly by Bichat and Parry, who were, however, probably misled by the result of their experiments; a misfortune which has happened to not a few, and in this instance because they preferred trusting to observations made under unnatural circumstances, rather than to those which are constantly presenting themselves without any experiment at all. They found that, when arteries are exposed, they do not dilate, and therefore they concluded that it is not usual for them to dilate. Were a person, on opening the chest, to conclude that the lungs he saw collapsed were in their natural state, would he draw a fair conclusion? Exposed arteries in the living body remain contracted, for the same reason that they contract if cut, to avert injury.

If the senses are to be trusted, arteries do dilate in the usual state of the body, when not exposed: it is apparent in the pulse of superficial arteries; it is sensible in a finger surrounded by a ring. I have observed it as most evidently proved by the alternate rise and fall of matter collected in a deep ulcer in the groin, over the femoral artery. It is evident in the jet from a divided artery, and in the pulsation of one that is tied.

But I may be allowed to ask, if the elasticity of the arteries in the lateral direction is not employed, wherefore was it given? Are we warranted in believing that it exists without a purpose? and what is that purpose, if not that now assigned?

It is demonstrable that, without this elasticity, there would have been no equable flow of blood in the smaller vessels; but it must have been forced through them by sudden, violent, and probably injurious impulses.

From consideration of the structure of the heart, it is evident that a portion of time must be allowed for its cavities to fill, as well as to empty themselves: that portion of time is considerably the longest, and consequently the blood can only leave it by sudden impulse. This sudden impulse, violent in itself, would be succeeded by a state of quiescence of the blood, (which we can prove does not exist,) but for this provision in the lateral elasticity of the arteries, which, like the main-spring of a watch, receives the force of the heart during its short exertion, and gradually parts with it again; so that the force so derived from the heart is really acting while the viscus itself is filling, and no interval occurs when it is not employed in propelling the blood.

A great deal has been said on a point to which I deem it necessary to advert, namely, whether the blood flows through the arteries in waves. I have above stated that the arguments predominate against its flowing merely in column, (particularly the fact, that on such a supposition there would be no continued stream from a cut artery, which it is well known there is,) but does it flow in waves, as has been repeatedly asserted? I should think not; for the supposition will hardly account for the instantaneous impression felt at each beat of the heart in arteries remote from it.

Between these two theories it is not impossible to find out an intermediate, which may approach to the truth. There can be little doubt that at each impulse of the heart a shock is diffused over the arterial system, which propels the blood in a longitudinal direction; but is it not quite clear that, whenever this is felt, there must also be an impulse on the sides of the tubes? and if so, these sides, being yielding in their nature, would yield or dilate; and if elastic, they would return to their former state, as the power ceases which dilates them. This hypothesis of an instantaneous dilatation of the arterial system, however, differs entirely from that of successive waves flowing along them like ordinary canals, and much more accords with the facts observed.

The elasticity of the arteries lessens, as might be expected, as they become more remote from the direct impulse of the heart.

The arteries, in addition to their elasticity, it is well known possess a contractile power. This cannot be doubted: it only remains to inquire how this power is exerted.

It has been repeatedly asserted by men of high authority, that this power is employed in supporting the circulation. This assertion does not readily admit of being directly disproved; but to the arguments which have been advanced against it, and which are not perhaps very conclusive, I will add the following:—If the hypothesis were true, it follows either that the contractile power must be exerted at the same moment throughout, or irregularly: if the latter, it must put an end to all uniformity in the circulation. If at the same moment throughout, then it must either correspond with the systole of the heart, or not; if it corresponded with the systole, then it would prevent the admission of blood from the heart, and in no way tend to forward it: if not, it would follow, I conceive, that this fresh impulse, most powerful certainly as the contractility increases,—i. e. as the arteries diminish in size,—would generate a new jet of blood through the subsequent channels; which is not the case, and therefore it may be concluded that it does not exist.

What, then, is the use of this contractile power of the arteries? I should answer, to regulate the supply of blood. It opens or shuts these canals more or less, according to the demand made by the extreme vessels, just as the intelligent hand of the engineer opens or closes the valves of pipes which supply the different quarters of a city more or less according to their wants: hence the arteries going to a part are obliterated after it is removed; hence, if a part is increasing with more than ordinary vigor, all the branch pipes leading to it enlarge: they will enlarge on the instant if leading to parts of temporary activity, as the salivary, mammary glands, &c. and will contract with the same speed. They will contract to prevent hemorrhage, or enlarge to supply parts whose regular supply has been interrupted.

Such is the degree to which this contractile power can be exerted, that it can instantaneously close an artery which is divided; and these vessels close upon the last portions of blood in the act of death,—at least, so it appears to me. Dr. Arnott has called this point in question; but, in the first place, I am by no means sure that we are warranted in believing, with him, that the vessels, so called capillaries, do in point of fact exert the powers of capillary tubes, as he states; and in the second, I do not conceive that the flatness of the arteries after death is a conclusive argument, as he infers, against their having emptied themselves: although found in a flat state after death, they may have been cylindrical during the existence of contraction, and subsequently become flat when the contractile power has ceased, and the pressure of superincumbent parts has acted upon them. The capillaries of the lungs do not exhaust the pulmonary artery, which they would do, as well as the systemic capillaries the aorta, if capillary attraction produced that phenomenon. The real cause of the difference would take too long a time here to discuss.

No doubt the contractile force of the arteries is in constant exertion, not only in varying the size of the arteries, as may be required, but as maintaining a degree of resistance which regulates the admission of the blood into them at every systole of the heart, and which mainly contributes to produce many of the qualities of the pulse; as, for instance, its size, hardness, &c. &c., which will result from the combined influence of the force of the heart, and the greater or less resistance of the arteries, which resistance will be uniform as respects their elasticity, variable as regards their contractile power. I cannot, however, think that this resistance places them in the condition of rigid tubes, as has been asserted, or that consequently the circulation can be calculated upon data which might be allowed if this fundamental hypothesis were true.

The blood having quitted the arteries, returns through the capillaries and veins to the heart: we are next to inquire how this is effected.

The force of the heart, though lessened, cannot be considered as by any means expended when the blood has reached the capillaries; and that it will still materially support the circulation, there can be no reason to doubt. Dr. Arnott* states, page 495, that "the heart keeps up a tension or pressure on the arteries of about four pounds per square inch of their surface, and with this force, therefore, is propelling the blood into the capillaries." Now, if it be granted that the amount of arterial pressure is correctly calculated, there are many facts which seem to show that there is some error in the application; more particularly the diminishing momentum of the blood in the smaller arteries, which may, perhaps, be more justly considered as the true measure of the force in them derived from the heart, than that founded on calculations, such as those which arise from the general tension of the arterial system.

If a number of holes of different sizes be made at the same height in a vessel of water, allowance being made for the friction of the sides of the holes, water will flow through these holes in streams of equal velocity; but if we regard the streams of blood flowing from the amputated surface of a limb, we find that the velocity of the streams diminishes nearly as the size of the arteries, proving, I think, that it is not from an equal degree of tension, or pressure, or force that they flow. Again, while considering this subject, I cannot help observing that the calculations of Dr. Hales, and other investigators, may have been erroneously deduced, inasmuch

* I cannot allude to Dr. Arnott's work without expressing my belief that it will prove exceedingly useful, and that the medical profession in particular is much indebted to him.

as they calculate for the whole, from the force which they see belongs to a stream proceeding from one open artery, without taking into consideration that the universal sympathy of the vessels will throw the blood upon that point; and, if so, it cannot be taken as a fair measure of the ordinary force in such an artery. If this opinion is true, it would probably be found that opening other arteries during such experiment would lessen the force of the stream in a greater ratio than, upon Dr. Hale's calculations, it ought to do.

That the propelling power of the heart, however, is strongly exerted in support of the circulation through the capillaries and veins, is pretty evident, notwithstanding Bichat's opinion to the contrary; but that it is not the sole agent may be deduced from the consideration of many facts, and one of these, which is of an anatomical nature, appears to me of considerable weight. The veins of the lower extremities are much stronger than those of the upper, manifestly because they have to support a longer column of blood. Now, if the sole force which propelled this blood was derived through the arteries, might we not expect a proportionate difference in *their* strength also? whereas, between the thickness of the coats of the arteries and veins of the lower extremities little comparative difference can be perceived.

It may be very difficult to measure the precise degree of influence which ought to be assigned to the heart, and what to the capillaries; but the following phenomena, which have been often quoted, all prove that they possess powers.

1st. Circulation in animals who have no hearts.

2d. Circulation in the human fœtus without hearts.

3d. Circulation in the human subject during syncope, &c.

4th. Bleeding after apparent death, and the heart's action has ceased.

5th. The circulation of the porta, spleen, corpora cavernosa, placenta, &c.

6th. The progress of fluids in the lacteals and lymphatics, and in the excretory ducts of the testis and other glands.

To these I may add the following—

In cases of obstruction of the orifice of the aorta, when the aperture has been reduced to the size of a crow-quill, and arterial pulsation has scarcely been perceptible, I have seen veins bleed most freely, not surely from the heart's action.

If a tourniquet be tightly screwed so as to compress every artery of a limb, the veins, when cut, will bleed freely, and be completely emptied.

In ordinary bleeding, blood will not flow from a vein, of course, if none is admitted into a limb; but if there is, the strength with which it flows by no means corresponds, in many cases, with the force of the arterial pulsation.

These facts, however, only prove the power the capillaries have of propelling the blood; they do not prove that the circulation in the veins is influenced by them in common; but they render it extremely probable, and it is very easy to conceive that, if a number of these vessels are every moment discharging their contents into the veins, tubes which are constantly contracting the areas of their diameters as they meet; that such a force will be generated in the larger branches as will materially assist the venous circulation.

It has always been deemed necessary to assign some auxiliary causes, besides the propulsive force of the heart, to explain in a satisfactory way this venous circulation: of these, there are many so manifestly incapable of aiding it, that it is only astonishing that men of high talent should for a moment have deemed them worthy of notice. Such are, the increasing size of the veins facilitating the return of blood; which is manifestly erroneous; as the sum of the branches is, on the contrary, much greater than the sum of the trunks;—the greater size of the veins than of the arteries; this would, if any thing, in the upright posture of the body, be a disadvantage rather than otherwise, and can only facilitate the return from the larger half of the body when we are in a horizontal posture. The valves, which may and do prevent a reflux of the blood, but never can aid its advance, except during muscular action, when an influence is created similar to that of the forcing pump: the action of muscles, however, is only occasional, and we cannot admit an occasional cause as productive of a permanent effect. The pulsation of contiguous arteries has also been held by Bichat to be another means; but it is extraordinary that he should not have perceived that this must act equally in each direction, and consequently retard as much as it propels.

Two other hypotheses remain, which deserve very different consideration. I mean the influence of the thorax, or of the heart, in sucking in the blood, particularly the latter.

It strikes me to be an insuperable objection to the former, that if, during respiration, a sucking power were exerted in the columns of blood approaching the heart, it must equally act on those which are quitting it, and consequently it would retard the arterial circulation as much as it assisted the venous. It does not, however, demand much argument to refute an hypothesis which is now little entertained, and which, at all events, fails to explain the phenomena of the circulation in whole classes of animals, whose blood is ventilated without any motions of a thorax.

The hypothesis which attributes to the heart a power of suction as well as propulsion, has of late, I need not say, much engaged the attention of very able physiologists, who have supported or opposed it with much ability and perseverance. Some of the arguments stated in Dr. Arnott's work against it appear to me very conclusive, particularly that derived from the well-known fact that blood flows with great force from a vein below a ligature, which must of course intercept any suction power of the heart.

Dr. Arnott's experiment, page 510, with respect to the impossibility of drawing up a column of fluid through a tube immersed in a bucket of water, appears to me to fail in an important particular,—namely, that the fluids in the veins may be supposed to be maintained in equilibrio by that in the arteries; whereas, in the experiment stated by Dr. Arnott, there is no counterpoise provided for, and the cases, consequently, have not a just and perfect analogy.

There is, however, an important fact connected with the structure of the heart, which I think goes far to prove the impossibility of the theory,—namely, the extreme disparity in the power of the right and left sides. Now, if the return of blood through the veins were effected by the suction of the heart, we might not unfairly conclude that an equal degree of force would be exerted in drawing as in propelling it, as the approaching column is, in point of fact, larger than that which is quitting the heart; and, upon this supposition, it would also follow that, if equal work is to be done, equal strength would appear in the structure, as in truth is the case when equal work is done by both sides of the heart, as is evinced by the comparative strength of the right ventricle during the fœtal state, and when the septum remains open from malformation. But what is the usual relative strength of the two sides? Instead of being at all equal, it is just that which we may well suppose would correspond with the relative efforts on either side to propel the blood through the pulmonic arteries and general system respectively.

There is another consideration which may be strongly urged; namely, that on the hypothesis of the heart exerting a suction power, not the least interval of relaxation would be allowed for its fibres, which must commence the process of dilatation the moment they had completed that of contraction; a phenomenon so entirely opposed to all we know of the principles of muscular power and mode of action, that it would go far to make me doubt an hypothesis which must assume a fact so irreconcilable.

To conclude: if it be urged that my arguments do not rest on the basis of experiment, I must beg leave to say that it by no means follows that they are on that account less entitled to credit, if they are grounded upon accurate observation of natural phenomena. Experiments, after all, are but an auxiliary, though a valuable auxiliary, in our pursuit of truth, and they serve only to supply that information which our observations cannot deduce from natural phenomena; and, if the conclusions drawn from them are evidently opposed to those which do arise from the consideration of natural phenomena, we have the strongest grounds for doubting their accuracy.

In the sciences, experiments have led to error as well as to true knowledge, man not having always rightly interpreted them; which has been repeatedly shown, but in no way more remarkably than in this, that learned men have drawn different—often opposite, deductions from the same experiments.

That the knowledge resulting from the observation of natural phenomena is quite as much entitled to belief as that from experimental research, is proved by nothing more strongly than the fact that a large proportion of physical truths rest upon no other foundation, especially in the department of astronomy and optics, where experiments are, indeed, almost wholly out of the question. That the same reasoning will apply to the science of the animal economy, I feel fully convinced; and, as a strong example of the truth of this position, I may express my persuasion that attentive observation alone of all the phenomena which present themselves relative to the flow of blood during hemorrhage or otherwise, aided by the careful consideration of the structure of the heart and vessels, might have enabled Harvey to establish precisely the same conclusions respecting the course of the circulation as those which he arrived at by experiment; and possibly he would have done so, had he commenced the inquiry free from the prejudices which at that time incumbered the investigation.

*Exeter; December 6th,* 1827.

