# Erratum

**Published:** 1828-02

**Authors:** 


**ERRATUM.**

In our last Number, for Dr. JAMES, read Mr. JAMES.

